# A time-frequency denoising method for single-channel event-related EEG

**DOI:** 10.3389/fnins.2022.991136

**Published:** 2022-11-25

**Authors:** Wenqiang Yan, Yongcheng Wu

**Affiliations:** School of Mechanical Engineering, Xi’an Jiaotong University, Xi’an, China

**Keywords:** event-related potential (ERP), bidimensional empirical mode decomposition (BEMD), non-local means (NLM), time-frequency denoising, signal processing

## Abstract

**Introduction:**

Electroencephalogram (EEG) acquisition is easily affected by various noises, including those from electrocardiogram (ECG), electrooculogram (EOG), and electromyogram (EMG). Because noise interference can significantly limit the study and analysis of brain signals, there is a significant need for the development of improved methods to remove this interference for more accurate measurement of EEG signals.

**Methods:**

Based on the non-linear and non-stationary characteristics of brain signals, a strategy was developed to denoise brain signals using a time-frequency denoising algorithm framework of short-time Fourier transform (STFT), bidimensional empirical mode decomposition (BEMD), and non-local means (NLM). Time-frequency analysis can reveal the signal frequency component and its evolution process, allowing the elimination of noise according to the signal and noise distribution. BEMD can be used to decompose the time-frequency signals into sub-time-frequency signals for noise removal at different scales. NLM relies on structural self-similarity to locally smooth an image to remove noise and restore its main geometric structure, making this method appropriate for time-frequency signal denoising.

**Results:**

The experimental results show that the proposed method can effectively suppress the high-frequency components of brain signals, resulting in a smoother brain signal waveform after denoising. The correlation coefficient of the reference signal, a superposition average of multiple trial signals, and the original single trial signal was determined, and then correlation coefficients were calculated between the reference signal and single trial signals processed by time-frequency denoising, ensemble empirical mode decomposition (EEMD)-independent component analysis (ICA), EEMD-canonical correlation analysis (CCA), and wavelet threshold denoising methods. The correlation coefficient was highest for the signal processed by the time-frequency denoising method and the reference signal, indicating that the single trial signal after time-frequency denoising was most similar to the waveform of the reference signal and suggesting this is a feasible strategy to effectively reduce noise and more accurately determine signals.

**Discussion:**

The proposed time-frequency denoising method exhibits excellent performance with promising potential for practical application.

## Introduction

Electroencephalogram (EEG) is used to measure the synchronous changes of postsynaptic potential produced by pyramidal neurons with similar orientation in the brain. Almost all sensory, motion, or mental events can cause transient changes in spontaneous EEG activity, for time locked and phase locked event-related potential (ERP) (Mouraux and Iannetti, 2008). Currently, the cross-trial averaging method is the most widely used method to detect event-related brain response. This method requires that event-related brain response signals are stable and unchanged in different trials, specifically, that the latency, amplitude, and waveform remain unchanged. However, characterization of the phase-locked ERP response revealed independent variation in the latency, amplitude, and waveform in different trials. Use of a cross-trial averaging method can improve the signal noise ratio (SNR) of brain response signals, but this approach result in the loss of information about cross-trial dynamic variation of event-related brain response signals. Overall, it is necessary to accurately estimate event-related brain response signals and their characteristics at the single-trial level. This approach can’t only help us to better analyze EEG data, but can also help exploration of the physiological and psychological functions of brain response signals. The amplitudes of almost all event-related brain response signals are far less than the amplitude of spontaneous EEG activity, and other physiological signals or non-physiological signals such as spatial electromagnetic noise can interfere with these signals (Ting et al., 2014; Huang et al., 2015; Haider et al., 2019). This interference increase the difficulty of reading EEG signals, thus impeding research, analysis, and application of these important brain function signals. Therefore, the development of advanced methods to remove noise interference from EEG signals and effectively improve the SNR of event-related brain response signals is a critical challenge for single-trial analysis and feature extraction.

The regression method is a traditional EEG denoising technique (Hillyard and Galambos, 1970; Whitton et al., 1978; Klados et al., 2011). Its basic assumption is that EEG and various noise components are additively superimposed (Sweeney et al., 2012). The regression analysis first defines the amplitude relationship between the reference channel and the EEG channel by the transmission factor, and then subtracts the estimated artifacts from the EEG to obtain clean EEG data. However, the need for one or more good regression reference channels limit their capacity for removing noise in EEG (Urigüen and Garcia-Zapirain, 2015). Filtering method is another commonly used EEG noise elimination technology, which generally includes three types: adaptive filtering, Wiener filtering and sparse decomposition. Adaptive filtering method makes the denoised EEG signal close to the reference signal by estimating the filtering parameters (Marque et al., 2005). However, adaptive filtering method is difficult to deal with sudden noise, such as some electromyography and continuous vibration artifacts (He et al., 2004). The idea of Wiener filtering is to minimize the power spectral density of the target signal and the measured signal (Somers et al., 2018). The problem is that the power spectral density of the target signal is generally unknown (Izzetoglu et al., 2005), which makes it difficult for Wiener filtering to be used for online filtering of EEG signals. The idea of sparse decomposition denoising is to sparsely represent the noisy signal on the over-complete atomic library, reconstruct the original signal with only several large representation coefficients, and shield the noise components contained in some small coefficients, so as to realize the denoising of the signal (Donoho, 2001; Li et al., 2013, 2014).

Blind source separation (BSS) is the most well-known method in EEG denoising field. This method can estimate the mixed signal without prior information (or with little information) of the source signal and during the mixing process. BSS has been widely used in the processing and analysis of EEG signals (Roy and Shukla, 2019; Zou et al., 2020; Desjardins et al., 2021). Independent component analysis (ICA) and canonical correlation analysis (CCA) are two classical BSS methods. Raw EEG data collected from the scalp are the sum of signals and artifacts. Signals and artifacts are considered to be independent of each other, and ICA is considered to be an effective method for artifact separation. ICA decomposes multi-channel observation signals into several independent components through an optimization algorithm that assumes the statistical independence of source signals. After the decomposition of original EEG data by ICA, the separated independent components can be divided into artifact-related and neural activity-related components. The artifact-related components are removed, and the other data are reconstructed data that should retain the real EEG signal. Due to its high efficiency in BSS, ICA has been widely used in steps of EEG signal processing, including noise cancelation, ERP component extraction, and single trial ERP analysis (Mcmenamin et al., 2011; Urigüen and Garcia-Zapirain, 2015; Lee et al., 2016). CCA is another effective method for BSS. Using the original EEG as the first data set and the time-delayed version as the second data set, CCA utilizes second-order statistics to identify sources that are maximally autocorrelated and mutually uncorrelated. CCA then selects representative comprehensive indicators (linear combinations of variables) from two groups of random variables, and uses the correlation relationship of these indicators to represent the correlation relationship between the original two groups of variables. Compared with the true EEG signal, an artifact signal exhibits a wider spectrum. This results in a relatively low autocorrelation, which is then used by CCA to isolate artifacts from EEG signals. Several studies have demonstrated the efficacy of CCA for artifact removal from EEG signals (Sweeney et al., 2013; Chen et al., 2014, 2016).

In addition to the above methods, the researchers also proposed a hybrid strategy combining signal decomposition and BSS to process EEG signals. Both ICA and CCA are multi-channel signal analysis methods. However, healthcare systems are evolving from hospital-centered to ambulation-based care, fewer channels are typically used for ambulatory EEG (Minguillon et al., 2017). Given this, there is significant interest in the development of single-channel techniques for EEG preprocessing. To use these methods for single-channel signal analysis, the single-channel signal must first be decomposed into multi-dimensional signal components using wavelet transform or ensemble empirical mode decomposition (EEMD). Next, BSS methods (e.g., ICA and CCA) are used to further decompose the generated multi-dimensional signal components into meaningful sources. These approaches have been implemented in the proposed wavelet ICA (WICA) (Mammone et al., 2012) and EEMD-ICA (Mijovi et al., 2010) methods. EEMD is an adaptive decomposition method that can decompose a one-dimensional signal into several intrinsic mode functions (IMF) according to its characteristics. However, with no prior knowledge of the signal of interest, it is difficult to select the optimal wavelet for wavelet transform. A comparison between EEMD-ICA and WICA found EEMD-ICA performed best for separating mixed EEG, electromyogram (EMG), and electrocardiogram (ECG) signals under a single-channel scenario (Mijovi et al., 2010). In a recent study, Chen et al. proposed EEMD-CCA for the removal of artifacts from single-channel EEG signals, and demonstrating that EEMD-CCA is a more reliable approach than EEMD-ICA (Chen et al., 2014, 2016, 2018). In addition to EEMD-ICA and EEMD-CCA, wavelet threshold denoising method is also applied as a single-channel brain signal noise cancelation method.

EEG signals have typical non-linear and non-stationary characteristics, and it is difficult to obtain all the statistical characteristics of EEG signals from only the time or frequency domains. Time-frequency analysis provides s one-dimensional signal in the form of a bidimensional time-frequency density function to reveal the signal frequency component and its evolution process. This will allow the elimination of noise based on the distribution of signal and noise. The bidimensional time-frequency signal can also be analyzed as an image, and then artifacts in the EEG signal can be effectively removed using an advanced image denoising method. Therefore, the goal of this study was to design a time-frequency analysis method for single-channel EEG denoising.

Short time Fourier transform (STFT) is a common time-frequency analysis method with inverse transform, allowing the transformation of a denoised time-frequency signal into a time-domain signal for subsequent analysis. Here, STFT was used to transform a one-dimensional EEG signal into a bidimensional time-frequency signal, which can be analyzed as an image. Bidimensional empirical mode decomposition (BEMD) is an adaptive decomposition method for non-linear and non-stationary data and has been widely used for image enhancement and denoising (Liu and Chen, 2019). In this study, BEMD was used to decompose time-frequency signals, and each bidimensional intrinsic mode function (BIMF) obtained can be analyzed as sub-images. Non-local mean (NLM) is another effective image denoising technique (Arabi and Zaidi, 2020). In NLM, the pixels in the image do not exist in isolation, but the pixels at a single point are related to other pixels elsewhere. Similar pixels are not limited to a certain local area, and natural images may contain abundant redundant information. Therefore, image blocks that describe the structural features of the image can be used to search for similar blocks within the whole image, to maximally maintain the detail features of the image while denoising. The time-frequency signal describes the energy density or intensity of the signal at different times and frequencies. The energy density of both the effective signal and the noise are distributed over the whole timeline with a certain correlation and similarity between the energy density at a specific timespoints and other timespoints. Thus, the characteristics of time-frequency signal are highly consistent with the image characteristics required by NLM. Therefore, in this study, the obtained BIMFs were filtered by NLM to achieve noise cancelation at different scales. The BIMFs were then averaged after denoising to obtain the reconstructed time-frequency signal. The denoised EEG time domain signal can then be obtained as the inverse STFT of the reconstructed time-frequency signal.

This study proposes a novel denoising method for brain signals in the time-frequency domain and applies the image denoising method to the denoising of time-frequency signals. We compared the similarity of ERP waveforms processed by the proposed method, EEMD-ICA, EEMD-CCA and wavelet threshold denoising methods with the superposition average waveform of multiple trial signals. The results show that the ERP waveform processed by time-frequency denoising method is more similar to the waveform after multi-trial superposing and averaging, demonstrating the effectiveness of the proposed method for brain signal denoising. Additionally, this new approach to time-frequency analysis of brain signals has potential value for use in the denoising of other types of signals such as those producted by thermography, X-ray imaging, electrocardiography, electromyography, and others.

The organization of this study is as follows. In section “Materials and methods,” the data and methods used in this study are introduced. Section “Results” compares the denoising results of the proposed method, EEMD-ICA, EEMD-CCA, and wavelet threshold methods, followed by discussions and suggestions for future work in section “Discussion.” Finally, we conclude the work in section “Conclusion.”

## Materials and methods

### Data used in this study and data pre-processing

The ERP dataset used in this study is comprised of target image detection tasks, and the dataset is freely available at https://doi.org/10.6084/m9.figshare.12824771.v1. The stimulation was presented by a 24.5-inch liquid crystal display (LCD) monitor with a resolution of 1920 × 1080 pixels and a vertical refresh rate of 60 Hz. Street scene images were presented at 10 Hz (10 images per second) in the center of the screen within a 1200 × 800-pixel square. The images containing humans were regarded as target images and the subjects were asked to press keys immediately after they detected a target. The dataset includes 14 healthy subjects and the sample rate is 1000 Hz. For each subject, the experiment consisted of three blocks. Each block contained 56 target image stimulus trials. For more information about the dataset, please refer to Reference (Zheng et al., 2020).

The data obtained 1 s after target image stimulation were extracted as the data for analysis. Data preprocessing was performed as follows. The EEG data were first down-sampled to 250 Hz. Next, the data were band-pass filtered within 2–30 Hz. For the analysis of EEG characteristics, the EEG data were re-referenced to the average of all electrodes [i.e., common average reference (CAR) (Mcfarland et al., 1997)].

### Ensemble empirical mode decomposition-independent component analysis and ensemble empirical mode decomposition-canonical correlation analysis methods

#### Ensemble empirical mode decomposition

Based on instantaneous frequency analysis, Huang et al. proposed an empirical mode decomposition (EMD) method to decompose one-dimensional signal into a series of IMFs (Huang et al., 1998). However, “mode mixing” can occur during EMD decomposition, where, under some conditions, different time scales are classified as the same IMF or signals of the same time scale are cut into multiple different IMF. To address this problem, Wu et al. proposed EEMD, an improved EMD algorithm (Wu and Huang, 2009). EEMD adds white noise with uniform frequency distribution and zero mean value to the analysis signal. This provides even distribution of the extreme points of the whole frequency band of the signal, which effectively avoids the problem of sparse distribution of low-frequency components and dense distribution of high-frequency components in the signal, thus ensuring the time-domain continuity of each IMF and alleviating the mode mixing problem. The EEMD process of a signal *x*(*t*) can be described as follows:

(1) Add a series of random white Gaussian noise *n*(*t*) with normal distribution and constant variance to the one-dimensional observation signal *x*(*t*) :


(1) $$x^{\prime }\,\left(t\right)=x\,\left(t\right)+n\,\left(t\right)$$

(2) Decompose the noise-added signal *x’*(*t*) into IMFs using the EMD method.


(2) $$x^{\prime }\,\left(t\right)=\sum_{i=1}^{k}c_{i,j}\,\left(t\right)+r_{j}\,\left(t\right)$$

where *c*_*i*_*_*j*_* is the *i*th IMF obtained by the *j*th decomposition, and *r*_*j*_(*t*) is the residue obtained by the *j*th decomposition.

(3) Repeat steps (1) and (2) *l* times, and add new random Gaussian white noise each time.

(4) Calculate the ensemble mean of the corresponding IMF of each decomposition, and obtain final IMFs:


(3) $$c_{i}^{\prime }\,\left(t\right)=\sum_{j=1}^{l}c_{i,j}\,\left(t\right)$$

(4) $$r^{\prime }\,\left(t\right)=\sum_{j=1}^{l}r_{j}\,\left(t\right)$$

After EEMD decomposition, the observation signal *x*(*t*) can be expressed as the sum of multiple IMFs:


(5) $$x\,\left(t\right)=\sum_{i=1}^{k}c_{i}^{\prime }+r^{\prime }\,\left(t\right)$$

A multichannel signal *X*(*t*) = [*c*_1_’, *c*_2_’,…, *c*_*k*_’, *r*’(*t*)] can be reconstructed by multiple IMFs and final residuals *r’*(*t*) of the one-dimensional signal *x*(*t*) decomposed by EEMD. BSS algorithms (i.e., ICA and CCA) suitable for multi-channel analysis can then be applied to the reconstructed signal *X*(*t*).

#### Independent component analysis

Independent component analysis is a method based on higher-order statistics, and it can be described as:


(6) X⁢(t)=A⁢S⁢(t)

Where *X*(*t*) = [*x*_1_(*t*), *x*_2_(*t*),…, *x*_*n*_(*t*)] is the *n*-dimensional observation signal, *S*(*t*) = [*s*_1_(*t*),*s*_2_(*t*),…,*s*_*m*_(*t*)] is the *m*-dimensional source signal, and *A* is the unknown signal mixing matrix. The goal of BSS is to recover the unknown *m*-dimensional source signal (*n* >*m*) from the *n*-dimensional observation signal. Equation (6) can be rewritten as:


(7) $$S^{\prime }\,\left(t\right)=W\,X\,\left(t\right)=W\,A\,S\,\left(t\right)$$

Independent component analysis is applied to find the reversible separation matrix *W*, and then the output source *S*’ can be obtained by a linear transformation on the signal *X*(*t*). Many objective function construction methods could be used to estimate the source signal by ICA. The fixed-point algorithm based on negative entropy maximization (FastICA algorithm) has fast convergence speed and high precision, so this method was selected for use in this study.

#### Canonical correlation analysis

Canonical correlation analysis is a statistical method that is used to study the linear relationship between two groups of multi-dimensional variables. Let *X*_1_(*t*) be the observed data matrix *X*(*t*) with *n* channels and *T* temporal samples, and let *X*_2_(*t*) be a temporally delayed version of the original data matrix *X*_2_(*t*) = *X*(*t*-1). Using the two sets of signals *X*_1_ and *X*_2_, CCA then finds two linear projection vectors *W*_1_ and *W*_2_ so that the two groups of linear combination signals *W*_1_*^T^ X*_1_ and *W*_2_*^T^ X*_2_ have the largest correlation coefficients. This leads to the following objective function that maximizes the correlation between the linear combinations of the components in *X*_1_ and *X*_2_:


(8) $$m\,a\,x_{W\,1,W\,2}\,\frac{W_{1}^{T}\,X_{1}\,X_{2}^{T}\,W_{2}}{\sqrt{W_{1}^{T}\,X_{1}\,X_{1}^{T}\,W_{1}}\,\sqrt{W_{2}^{T}\,X_{2}\,X_{2}^{T}\,W_{2}}}$$

Equation (8) can be solved by constructing Lagrange function and performing eigenvalue decomposition. The typical correlation variables can be calculated by solving the projection vector:


(9) V=WX1T1

The dimension of canonical correlation variable *V* is *n × T*, where the first line is called the first canonical correlation variable, or the component that can best represent the set of signal characteristics. In this study, the first canonical correlation variable was selected as the signal denoised by CCA.

#### Ensemble empirical mode decomposition-independent component analysis

A multichannel signal, *X*(*t*) ∈ *n* × *T*, is reconstructed from IMFs and residue signals and used as the input to the FastICA, so ICA can decomposed *X*(*t*) into multiple independent source components. The source components obtained from ICA decomposition usually require further analysis to screen out effective signal components from the source components. In this study, the performance of the denoising algorithm was evaluated by comparing the similarity of the denoising signal waveform and the superposition average waveform of multiple trials. To do this, we calculated the similarity between the source components obtained by ICA decomposition and the multiple trial superposition average waveform, and then took the source component with the greatest similarity as the signal component after EEMD-ICA denoising.

#### Ensemble empirical mode decomposition-canonical correlation analysis

The multi-channel signal *X*(*t*) and its delay signal *X*(*t*-1) were, respectively, denoted as *X*_1_(*t*) and *X*_2_(*t*). *X*_1_(*t*) and *X*_2_(*t*) were taken as the inputs of the CCA algorithm. The canonical correlation variable *V* was calculated using the projection vector *W*_1_ as obtained by equation (8) and the first canonical correlation variable was taken as the signal denoised by EEMD-CCA.

### Wavelet threshold denoising method

The wavelet coefficients are generated by the observation signal *x*(*t*) through the wavelet transform and contain important information about the signal. The wavelet coefficients corresponding to the active component of the signal are relatively larger, while those corresponding to the noise component are relatively smaller, with smaller wavelet coefficients of the noise components than for the signal component. By selecting an appropriate threshold, wavelet coefficients larger than the threshold are retained and those below the threshold are considered generated by noise, so are set to zero for denoising. In this study, a one dimensional signal *x*(*t*) was denoised based on wavelet threshold by using the MATLAB library functions *ddencmp* and *wdenencmp*.

### Our proposed time-frequency denoising method

#### Short-time Fourier transform

EEG has typical non-linear and non-stationary characteristics, with a spectrum that changes greatly with time. A single dimension of time or frequency is not sufficient to effectively summarize the characteristics of the whole signal. Time-frequency analysis provides distribution information of both the time and frequency domains and clearly describes the relationship between signal frequency and time, which can be used to eliminate noise. Here, transforming the time domain signal to the time-frequency domain was applied for denoising. Because STFT has inverse transform and its calculation is simple, STFT was used to transform the one-dimensional observation signal *x*(*t*) into the two-dimensional time-frequency signal *X*(*t*, *f*). This can be regarded as an image for analysis, where *t* and *f* represent the time axis and frequency axis of the time-frequency signal, respectively.

#### Bi-dimensional empirical mode decomposition

Bi-dimensional empirical mode decomposition is an effective adaptive multi-scale analysis method that is appropriate for the analysis and processing of non-linear and non-stationary signals. Here, BEMD was used to decompose the time-frequency signal *X*(*t*, *f*) into sub-time-frequency signals at different scales. BEMD can adaptively decompose a two-dimensional signal into a set of bi-dimensional intrinsic mode functions (BIMFs) with a residue. In the first BIMF, the highest instantaneous frequency component or the highest local spatial scale is extracted, and in the last BIMF, the lowest local frequency component is extracted. The residue describes the longer period duration, which reflects the trend of the signals. The general procedure of the BEMD can be described as follows:

(1) Identify all extremum of the original 2D signal *X*(*t*, *f*) through field comparison;

(2) Utilize all extremum to construct the maximal envelope *e_*max*_* and the minimal envelope *e*_*min*_, and compute the envelope mean *E*_1_(*t*, *f*) using the following equation:


(10) $$E_{1}\,\left(t,f\right)=\frac{e_{m\,a\,x}+e_{m\,i\,n}}{2}$$

The cubic spline interpolation was used as a surface-fitting method.

(3) Compute the difference between *X*(*t*, *f*) and *E*_1_(*t*, *f*):


(11) $$h_{1}\,\left(t,f\right)=X\,\left(t,f\right)-E_{1}\,\left(t,f\right)$$

*h*_1_(*t*,*f*) is an intermediate calculated value. The above process is repeated *p* times until *h*_1,_*_*p*_*(*t*, *f*) meets the BIMF decomposition condition, where:


(12) $$h_{1,p}\,\left(t,f\right)=h_{1,\left(p-1\right)}\,\left(t,f\right)-E_{1}\,\left(t,f\right)$$

(4) Set *c*_1_(*t*, *f*) = *h*_1,_*_*p*_*(*t*, *f*), and *c*_1_(*t*, *f*) denotes the first separated BIMF. Then *c*_1_(*t*, *f*) can be separated from the original data to obtain the remainder *r*_1_(*t*, *f*):


(13) $$r_{1}\,\left(t,f\right)=X\,\left(t,f\right)-c_{1}\,\left(t,f\right)$$

(5) The residue *r*_1_(*t*,*f*) is then treated as the new data subject of the sifting process. The procedure is then repeated *N* times until the standard deviation is less than a predefined threshold:


(14) $$\sum_{t=0}^{X}\sum_{f=0}^{Y}\frac{{\left[h_{N,p-1}\,\left(t,f\right)-h_{N,p}\,\left(t,f\right)\right]}^{2}}{h_{N,p-1}^{2}\,\left(t,f\right)}<\varepsilon$$

where *X*,*Y* is the field size of *X*(*t*, *f*). The BEMD criterion to stop the sifting process is based on the residues of two consecutive BEMD processes. Thus, the value of ε determines the number and property of the BIMFs. In general, the threshold ε is set at 0.25.

(6) The sifting process allows the decomposition of the signal *X*(*t*, *f*) into *N* BIMFs and a residue as:


(15) $$X\,\left(t,f\right)=\sum_{i=1}^{N}B\,I\,M\,F_{i}\,\left(t,f\right)+r_{N}\,\left(t,f\right)$$

where *r*_*N*_(*t*,*f*) represents the overall trend of the data and the BIMFs are the decomposed detailed information. BIMFs and *r*_*N*_(*t*,*f*) can be regarded as sub-images for analysis.

#### Non-local means

The NLM method considers the self-similarity property of the image, and the estimated value of the current pixel in the image is obtained by the weighted average of the pixels with similar neighborhood structure. For time-frequency analysis, the time-frequency signal represents the evolution of signal energy density with time, and the energy density at the current moment is similar or correlated with that at other moments, which is consistent with the algorithm idea of NLM. Here, NLM filter was used to denoise BIMFs and *r*_*N*_(*t*, *f*). The NLM algorithm assumes that the noise model is:


(16) F=X+0N0

Where *X*_0_ is the original image, *N*_0_ is the Gaussian white noise with mean of 0 and variance of σ^2^, and *F* is the image polluted by noise. In this study, *F* is the BIMF and *r*_*N*_(*t*, *f*) is obtained by BEMD decomposition of time-frequency signal *X*(*t*, *f*). For a given noisy image:


(17) F={F⁢(i)|i∈I}

Where *I* represents the coordinate range of the whole image. For any pixel *F*(*i*) in *I*, NLM uses the weighted average of all pixels in the whole noisy image to obtain the estimated value of this pixel:


(18) $$N\,L\,F\,\left(i\right)=\sum_{j\in I}w\,\left(i,j\right)\,F\,\left(j\right)$$

The value of *w*(*i*, *j*) depends on the similarity between pixel *F*(*i*) and *F*(*j*), and this is measured by the Gaussian weighted Euclidean distance *d*(*i*, *j*) of the neighborhood matrices *N*(*i*) and *N*(*j*) centered on *F*(*i*) and *F*(*j*), and *d*(*i*, *j*) can be expressed as:


(19) $$d\,\left(i,j\right)={||N\,\left(i\right)-N\,\left(j\right)||}_{2,a}^{2}$$

*d*(*i*,*j*) represents the *L*^2^ norm of the Gaussian weighted distance between the domain matrices *N*(*i*) and *N*(*j*), and *a* is the standard deviation of the Gaussian function. In Gaussian weighting, the discrete Gaussian function template is used to weight the Euclidean distance. The pixels closer to the center have higher weight and the pixels farther from the center have lower weight. The weight *w*(*i*, *j*) is defined as:


(20) $$w\,\left(i,j\right)=\frac{1}{c\,\left(i\right)}\,f_{k}\,\left(d\,\left(i,j\right)\right)$$

(21) $$f_{k}=e\,x\,p\,\left(-\frac{d\,\left(i,j\right)}{h^{2}}\right)$$

(22) $$c\,\left(i\right)=\sum_{j\in I}e\,x\,p\,\left(-\frac{d\,\left(i,j\right)}{h^{2}}\right)$$

Where *c*(*i*) is the standardization coefficient, and the parameter *h* is the attenuation coefficient of the exponential function. The signal processed through equation (18) is the image signal after denoising.

#### Time–Frequency denoising algorithm

**FIGURE 1:**
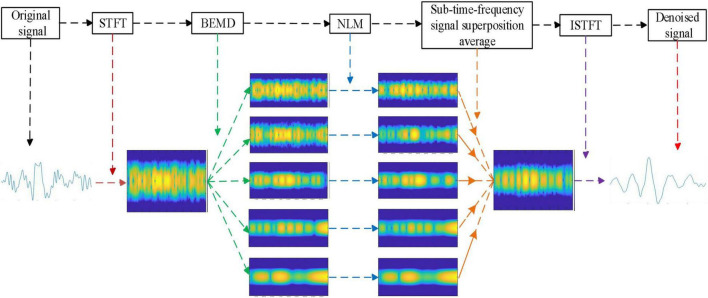
The time-frequency denoising algorithm flow.

Figure 1 shows the flowchart of the designed time-frequency denoising algorithm. The main steps of this algorithm are as follows:

(1) Performing STFT on the observation signal *x*(*t*) to obtain the time-frequency signal *X*(*t*, *f*);

(2) Decomposing *X*(*t*,*f*) by BEMD to obtain a series of BIMFs (including residue signals);

(3) Applying NLM filter to each BIMF;

(4) Superimposing and averaging the BIMFs processed by NLM to obtain the reconstructed time-frequency signal *X* ’(*t*, *f*);

(5) Obtaining the denoised time-frequency signal *x*’(*t*) by obtaining the inverse STFT on the time-frequency signal *X* ’(*t*, *f*).

## Results

### Analysis of modal decomposition results of time-frequency signals by bidimensional empirical mode decomposition

**FIGURE 2:**
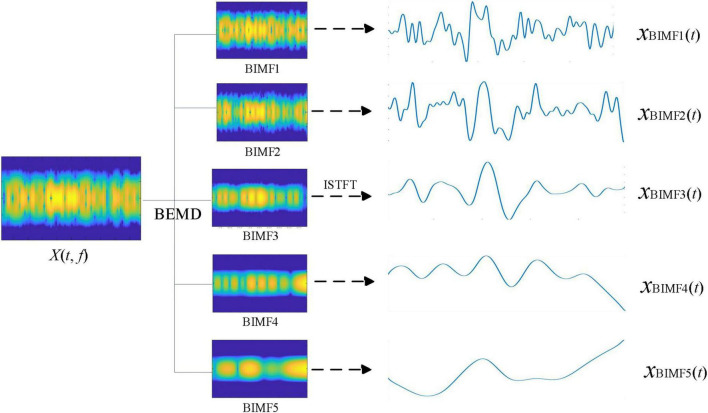
The modal decomposition results of time-frequency signals by bidimensional empirical mode decomposition (BEMD).

After transforming the observation signal *x*(*t*) into the time-frequency signal *X*(*t*, *f*), BEMD was used to decompose the time-frequency signal *X*(*t*, *f*). We then verified the successful decomposition of *X*(*t*, *f*) into sub-time-frequency signals of different scales by BEMD. Figure 2 shows the BEMD decomposition of the time-frequency signal *X*(*t*, *f*) into five BIMFs. The series of BIMFs time-frequency diagrams show that the signal frequency component from BIMF_1_ to BIMF_5_ gradually decreased. BIMF_1_ contained the highest signal frequency component and BIMF_5_ contained the lowest signal frequency component. Next, the inverse STFT was generated for individual BIMFs to observe the decomposition results of time-frequency signals by BEMD in the time domain. As shown in Figure 2, the frequency of the time-domain signal gradually decreased from *x*_BIMF1_(*t*) to *x*_BIMF5_(*t*), with the main components of the signal concentrated in *x*_BIMF1_(*t*) _‵_ *x*_BIMF2_(*t*) and *x*_BIMF3_(*t*). Clearly, *x*_BIMF5_(*t*) can be regarded as residue signal. This result indicates that BEMD can adaptively decompose EEG time-frequency signals into a series of sub-time-frequency signals of different scales. In the next step of analysis, denoising the sub-time-frequency signals can be performed to eliminate the noise in the time-frequency signals at different scales, thus enhancing the SNR of the event-related brain response signals.

### Performance analysis of sub time-frequency signal denoising by non-local means

**FIGURE 3:**
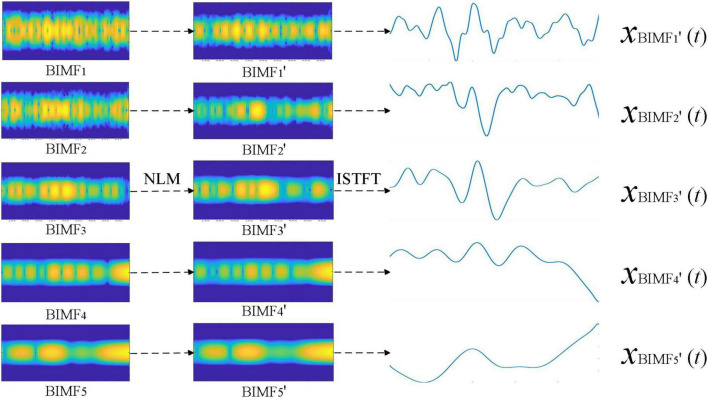
The denoise results of sub time-frequency signals by non-local means (NLM).

Non-local means was next used to denoise the BIMFs obtained by BEMD decomposition of the time-frequency signals *X*(*t*, *f*). We analyzed the denoise performance of NLM processing of BIMFs as shown in Figure 3. Comparison of the time-frequency signals for BIMF*_*i*_* and denoised signal BIMF*_*i*_* “show the greatest denoising effect of NLM on high-frequency components. After denoising, the energy was more concentrated for BIMF_1_,” “BIMF_2_,” and “BIMF_3_,” with good suppression of side band energy. BIMF_4_ “and BIMF_5_” represent the low frequency components, and exhibited little change compared with the original signals BIMF_4_ and BIMF_5_.

The inverse STFT of the denoised time-frequency signal BIMF*_*i*_*“was generated to evaluate the denoising result of NLM from the perspective of the time domain. The signal *x*_*BIMF*_*_*i*_*(*t*) shown in Figure 2 corresponds to the denoised signal *x*_*BIMF*_*_*i*_*” (*t*) presented in Figure 3. Compared with “*x*_*BIMF*_*_*i*_*(*t*) and *x*_*BIMF*_*_*i*_*”(*t*), NLM significantly denoised the high-frequency component signals [*x*_BIMF1_“(*t*) and *x*_BIMF2_” (*t*)]. After denoising, the time-domain waveform was smoother with good suppression of the high-frequency components. The low frequency components [*x*_*BIMF4*_“(*t*) and *x*_BIMF5_” (*t*)] exhibited little change compared with those before denoising.

**FIGURE 4:**
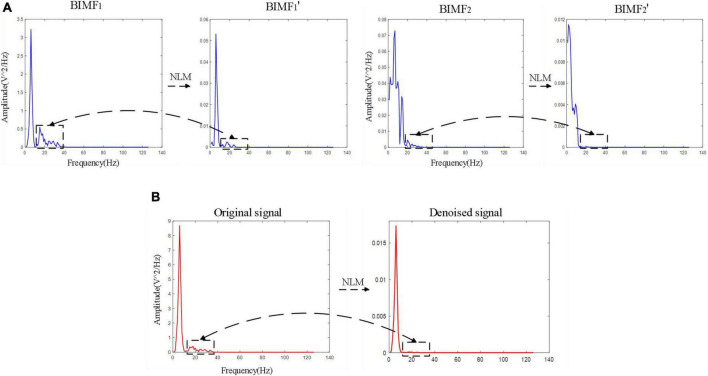
**(A)** The spectrum of BIMF_1_ and BIMF_2_ and their spectra after denoising by non-local means (NLM). **(B)** The spectrum of the original signal and the signal after time-frequency denoising.

The denoising results of NLM method were also analyzed from the perspective of frequency domain. Figure 4A shows the spectrum of BIMF_1_ and BIMF_2_ and their spectra after denoising by NLM. As shown, there were significant high-frequency interference components in BIMF_1_ and BIMF_2_ (indicated by the black box in Figure 4A), while the high-frequency components of BIMF_1_ and BIMF_2_ were significantly suppressed after NLM treatment. Figure 4B shows the spectrum of the original signal and the signal after time-frequency denoising. As can be seen from this comparison, the high-frequency component of the original signal was effectively eliminated after time-frequency denoising. The BIMFs decomposed by BEMD contain the local features of the original signal at different scales, where the high-order BIMF components represent the high-frequency components of the signal and the event-related brain response signals are mainly low-frequency components. These experimental results indicate that NLM significantly inhibits the high frequency components, so NLM application will enhance the low frequency components of the event-related brain signals for improved SNR of the ERP component of brain signals.

### Performance analysis of time-frequency denoising method

**FIGURE 5:**
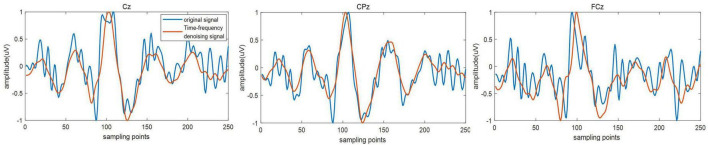
The original single trail signal and the signal after denoising.

As shown in Figure 2, among the five BIMFs decomposed from time-frequency signal *X*(*t*, *f*), the fifth BIMF contained almost no effective signal component and only represents the signal residue decomposed by BEMD. For this reason, the decomposition number of BEMD in the time-frequency denoising method was set to five, without further decomposition of the fifth BIMF. Figure 5 presents an original single trail signal and the signal after denoising of three electrode channels from a single subject (subject 2). As can be seen from the figure, the signal after time-frequency denoising effectively fit the trend of waveform of the original signal, the denoised signal was smoother, and the high-frequency component in the original signal was effectively suppressed. ERP refers to the positive or negative potential at a specific time after the appearance of the target stimulus. As can be seen from Figure 5, there was a lot of noise in the original signal, making it difficult to determine the effective ERP component. After time-frequency denoising, the variation trend of the time-domain waveform can more easily be observed. For example, the P300 component that appears in 300–400 ms was effectively enhanced.

Superimposing and averaging multiple trial signals is a common method to extract ERP components and effectively improve the SNR of ERP signals. We next compared the performance of the proposed time-frequency denoising method with the previously described methods of EEMD-ICA, EEMD-CCA, and wavelet threshold denoising. To do this, the superposition average of 56 trial signals was taken as the reference signal, and then 56 single trial signals were separately denoised by the time-frequency denoising method, EEMD-ICA, EEMD-CCA, and wavelet threshold method. The correlation coefficient between the denoised single trial signal and the reference signal was calculated, and the performance of the denoising method was measured by assessing the similarity between the denoised signal and the reference signal waveform.

**FIGURE 6:**
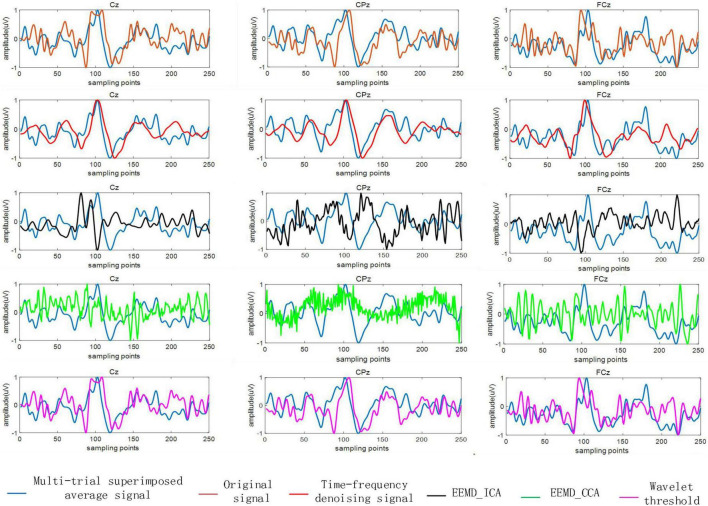
The reference signal and the signal after denoising by the time-frequency denoising method, ensemble empirical mode decomposition (EEMD)-independent component analysis (ICA), EEMD-canonical correlation analysis (CCA), and wavelet threshold method.

Figure 6 shows the analysis results of single trial signals in three electrode channels from a single subject (subject 2). Application of the EEMD-ICA method resulted in multiple source signals after ICA, and the source signals that are most relevant to the reference signal are shown in Figure 6. As shown, the performance of EEMD-CCA method was the worst. The signal processed by EEMD-CCA completely failed to fit the original signal waveform and differed greatly from the reference signal. The signal processed by EEMD-ICA was also quite different from the reference signal. The signal processed by the wavelet threshold method was similar to the original signal, but the high-frequency interference components in the original signal were not removed effectively. The single trial signal processed by the proposed time-frequency denoising method showed strong waveform similarity with the reference signal, with elimination of the high frequency component. The results indicate that the time-frequency denoising method is a more effective method for EEG denoising.

**FIGURE 7:**
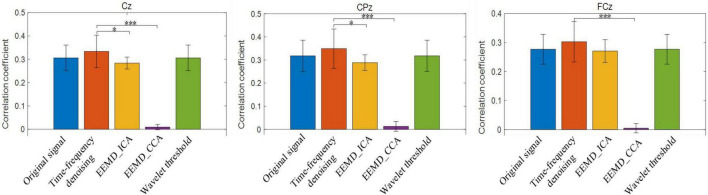
The average correlation coefficients between the original single trial signal and the reference signal and those between the single trial signal processed by time-frequency denoising, ensemble empirical mode decomposition (EEMD)-ICA, EEMD-canonical correlation analysis (CCA), and wavelet threshold methods and the reference signal. *p < 0.05, ^***^p < 0.0001.

In this study, the signal processing results of 56 single trials in all three blocks for each subject were superimposed and averaged, and then the experimental results of 14 subjects were superimposed and averaged. The mean correlation coefficients are shown in Figure 7. The time-frequency denoising method achieved the highest correlation coefficient for the three electrode channels, and the EEMD-CCA method exhibited the lowest correlation coefficient. The EEMD-ICA and wavelet threshold denoising methods had close correlation coefficients with the original signal. For CPz and Cz channels, the performance of the time-frequency denoising method significantly differed from that of EEMD-ICA and EEMD-CCA methods (*p < 0.05, ^***^p < 0.0001).

Both ICA and CCA are applicable to multi-channel signal analysis, and there is a strong correlation between each channel. However, each IMF decomposed by EEMD belongs to a different time scale, with only weak correlation between signals of each scale. This may be why EEMD-ICA and EEMD-CCA do not achieve effective noise elimination. Additionally, the short length of the analyzed data may also explain the lower performance of EEMD-ICA and EEMD-CCA methods. EEMD-CCA is used to analyze the canonical correlation between the original signal and its delay signal, and CCA is based on Pearson correlation. However, because the ERP signal is highly correlated with time, the delay will lead to the change of signal phase and the shift of signal phase will lead to a sharp decline of the Pearson correlation coefficient. Overall, constructing new signals by time delay for CCA analysis cannot enhance the correlation of the active components of signals, and may even play a negative role. This may explain why the EEMD-CCA method fails to achieve an effective denoising effect for ERP signals. In conclusion, the above results indicate that EEG signals processed using this new time-frequency denoising method exhibit a higher similarity to the reference signals, demonstrating that the proposed method allows better denoising of event-related brain response signals.

## Discussion

The accurate estimation of event-related brain response signals and their characteristics at the single trial level is required to analyze EEG data and also to further explore the physiological and psychological functions of brain response signals (Tu et al., 2016). There is a significant interest in improved analysis of single-channel signals for several reasons. First, with the continuous development of science and technology, the medical system has shifted from traditional hospital-centered care to mobile phone-based systems. For greater mobility, EEG collection devices are getting smaller and smaller, and some devices utilize only a single channel. Second, single-channel signal analysis is the basis of analysis of multi-channel signals and brain functional networks, and the effective tools for single-channel analysis can also be applied to the analysis of multi-channel signals. EEG signals are typically very weak and subject to noise interference (such as noise from ECG, ECG, EMG, motion artifacts, and others), so the development of advanced strategies for the denoising of single-channel signals has become an important research topic.

The human brain is a complex non-linear system, and EEG signals are also non-linear, varying in space and time. As a physiological signal, EEG has a relatively small range of potential change (microvolt level), a very fast speed of change, strong randomness, and large noise and background interference. For signals with non-linear and non-stationary characteristics such as EEG, it is necessary not only to pay attention to the frequency of the signal, but also to characterize the frequency of the signal at different times. Overall, the analysis of EEG signals in the time-frequency domain is more consistent with the characteristics of brain signals. The goal of this work was to address the problem of strong noise interference of single-channel signals by application of an effective time-frequency noise elimination method.

We propose a new adaptive EEG signal denoising method by converting a one-dimensional EEG signal to a two-dimensional one from images in the time-frequency domains using STFT, BEMD, and NLM approaches. Applying inverse transform of STFT, the denoised time-frequency signal can be transformed into the time domain for the time-frequency analysis of a single channel signal. Before denoising the two-dimensional time-frequency signal, BEMD was used to decompose the time-frequency signal. BEMD is appropriate for the analysis of non-linear and non-stationary signals. and can adaptively decompose the time-frequency signal into a series of BIMFs of different scales. The noise can then be eliminated at different scales by denoising the BIMFs. Our results verified the feasibility of decomposing time-frequency signals into sub-time-frequency signals of different scales by BEMD. The sub-time-frequency signal can be analyzed as an image, and we selected the NLM method to denoise the sub-image signal. According to NLM, images generally have the property of self-similarity, where pixels at different positions in the image often show strong correlation. NLM allows the local smoothing of an image based on structural self-similarity, resulting in the denoising and restoration of the main geometric structure of an image. The assumption of an image feature in NLM is also consistent with the feature of the time-frequency signal. The time-frequency distribution of this kind of signal reflects the evolution of the signal energy density with time, where the energy density at the current time point is correlated with the energy density at other timespoints, making NLM an appropriate tool for time-frequency signal denoising. We tested the denoising of time-frequency signals by NLM and observed an obvious inhibitory effect on the high-frequency component of the signals, but little effect on EEG signals in the low frequency band (i.e., the effective EEG signal frequency band), indicating that NLM can effectively denoise time-frequency signals. Analysis of the performance of the time-frequency denoising method showed that the waveform variation trend of the signal after time-frequency denoising fit well to the original signal, exhibited a smoother waveform, and effectively suppressed the high frequency component. Using the superposition average of multiple trial signals as the reference signal, the correlation coefficient was calculated between the reference signal and the original single trial signal, and between the reference signal and single trial signals after processing by the time-frequency denoising method, EEMD-ICA, EEMD-CCA, and wavelet threshold denoising methods. The results show the highest correlation coefficient between the signal processed by the time-frequency denoising method and the reference signal, indicating that the single trial signal after application of this time-frequency denoising method exhibited highest similarity to the waveform of the reference signal.

In summary, the time-frequency denoising method proposed in this study exhibits outstanding performance. There are several potential directions for future work. For example, time-frequency signals can be analyzed as images. Previous studies proposed many effective image denoising methods (Elad and Aharon, 2006; Dabov et al., 2007), and deep learning methods continue to provide advances in the field of image analysis (Kai et al., 2017; Zhang et al., 2018). All of these methods can be tested for analysis of time-frequency signals. Since time-frequency analysis may be appropriate for the non-linear and non-stationary characteristics of brain signals, feature extraction and feature classification of brain signals should also be considered in time-frequency domain. Spatial filtering may also improve brain signals analysis. After transforming multi-channel brain signals into time-frequency domain, an effective time-frequency signal fusion method can be developed to extract the characteristics of multi-channel brain signals. Overall, time-frequency analysis of brain signals has broad application prospects and future work should explore use of multiple strategies to decrease noise and improve SNR.

## Conclusion

To address the issues of strong noise interference and weak features of brain signals that limit effective study and interpretation of brain signals, a time-frequency denoising algorithm framework combining STFT, BEMD, and NLM was designed based on the non-linear and non-stationary characteristics of brain signals. In this algorithm, BEMD is used to decompose time-frequency signals into sub-time-frequency signals of different scales, and NLM method is used to effectively suppress the high-frequency components of signals for noise elimination at different scales. The experimental results show that the time-domain waveform of the brain signals after STFT-BEMD-NLM processing well fits the trend of the original signal waveform, and the waveform is smoother. We compared the correlation coefficients between the original single trial signal and the reference signal obtained by superposing and averaging multiple trial signals and those between the single trial signal processed by time-frequency denoising, EEMD-ICA, EEMD-CCA, and wavelet threshold methods and the reference signal. The results show that the brain signal after processing by the proposed method is most similar to the waveform of the reference signal, indicating that the proposed time-frequency method has better denoising performance and can be considered for practical brain signal analysis and processing.

## Data availability statement

The datasets presented in this study can be found in online repositories. The names of the repository/repositories and accession number(s) can be found below: https://doi.org/10.6084/m9.figshare.12824771.v1.

## Ethics statement

This study was approved by the Ethics Committee of Tsinghua University. The patients/participants provided their written informed consent to participate in this study. Written informed consent was obtained from the individual(s) for the publication of any potentially identifiable images or data included in this article.

## Author contributions

WY worked on the algorithm and wrote the manuscript. YW contributed discussions and suggestions throughout this project. Both authors contributed to the article and approved the submitted version.
